# Phytochemicals from *Turnera subulata* Exhibiting Antioxidant, Immunomodulatory, and Microbiological Activity

**DOI:** 10.3390/foods15111841

**Published:** 2026-05-22

**Authors:** Antonio Carlos Vital Júnior, Shênia Santos Monteiro, Genil Dantas de Oliveira, Yuri Mangueira do Nascimento, Fábio Miguel Santos Costa, Wêndeo Kennedy Costa, Alisson Macário de Oliveira, Maria Tereza dos Santos Correia, Daniele de Figueredo Silva, Rafael Wesley Bastos, Hugo Miguel Lisboa, Matheus Augusto de Bittencourt Pasquali

**Affiliations:** 1 Department of Biochemistry, Federal University of Rio Grande do Norte, Natal 59078-900, Brazil; ac.vitaljunior@outlook.com; 2 Department of Biochemistry, Federal University of Rio Grande do Sul, Porto Alegre 90035-003, Brazil; shenia-monteiro@hotmail.com; 3 Post Graduation Program in Bioactive Natural and Synthetic Products, Federal University of Paraíba, João Pessoa 58051-900, Brazil; 4 Department of Chemical Sciences, Faculty of Pharmacy, University of Porto, 4050-313 Porto, Portugal; fmscosta91@gmail.com; 5 Department of Biochemistry, Federal University of Pernambuco, Recife 50670-420, Brazilalisson.macario@ufpe.br (A.M.d.O.);; 6 Department of Microbiology and Parasitology, Federal University of Rio Grande do Norte, Natal 59078-900, Brazil; 7 Graduate Program in Food Engineering, Federal University of Campina Grande, Campina Grande 58429-900, Brazil

**Keywords:** *T. subulata*, aerial parts, hydroethanolic extract, phenolic compounds, redox behavior, cytokine modulation, *Staphylococcus aureus*, antimicrobial activity

## Abstract

*Turnera subulata* is traditionally used to treat inflammatory and infectious conditions; however; its biological activities remain incompletely characterized. In this study, aqueous (AETS) and hydroethanolic (HETS) extracts obtained from the aerial parts (leaves, stems, and flowers), as used in traditional infusions, were compared regarding physicochemical composition, redox behavior, cytotoxicity, immunomodulatory, and antimicrobial activities. HETS showed significantly higher phenolic content (2555.96 ± 43.55 mg GAE/100 mL) compared to AETS (1269.54 ± 20.60 mg GAE/100 mL) and exhibited stronger DPPH (83.05 ± 0.05%) and ABTS (85.1 ± 1.5%) radical scavenging activity. In contrast, AETS showed greater antioxidant capacity in the TRAP assay from 50 µg/mL (*p* < 0.0001). Both extracts displayed dose-dependent pro-oxidant behavior in the deoxyribose/Fenton system. In vitro assays demonstrated that both extracts exhibited dose-dependent cytotoxicity in SH-SY5Y cells, with no significant cytotoxic effects observed at concentrations ≤ 50 µg/mL. HETS significantly increased IL-10 levels (*p* < 0.05), indicating immunomodulatory activity. In antimicrobial assays, HETS showed selective activity against *Staphylococcus aureus*, with MIC values ranging from 0.625 to 1.25 mg/mL, while no relevant inhibition was observed against *Escherichia coli.* No synergistic interaction with vancomycin was detected. Overall, the results indicate that the extraction solvent strongly influences the phenolic enrichment and biological activity. The hydroethanol extract showed the most consistent bioactivity, highlighting its potential for applications as a natural antioxidant, immunomodulatory, and anti-staphylococcal agent. Future studies should focus on compound isolation, mechanistic validation, and evaluation in in vivo models to support potential commercial and therapeutic applications.

## 1. Introduction

Medicinal plants constitute one of the oldest sources of therapeutic agents used by humankind. Plant extracts play a central role in the treatment and prevention of diseases, due to their pharmacological properties and the wide availability of bioactive metabolites [1]. *T. subulata* is a medium-sized perennial shrub distributed throughout Africa and the Americas, with a notable occurrence in the northeast region of Brazil. In traditional medicine, this species is used to treat various conditions, including diabetes, respiratory infections, skin wounds, neoplasms, hypertension, gastrointestinal disorders, chronic pain, and inflammatory processes [2,3]. The bioactive properties of these species are often attributed to phenolic compounds, flavonoids, and alkaloids present in their extracts [4]. Despite its widespread use in folk practices, the number of systematic studies addressing the pharmacological and biological profiles of *T. subulata* remains limited, highlighting the need for further research to characterize its therapeutic potential.

The extracts of *T. subulata* are rich in secondary metabolites, such as tannins, saponins, and polyphenols [5]. Phenolic compounds are the main class of metabolites present in the genus Turnera. In extracts of *T. subulata*, three phenolic compounds are presented as valuable chemical constituents, namely vitexin-2-O-rhamnoside, 7-O-beta-glucopyranosyl-4′-hydroxyl-5-methoxyisoflavone, and ferulate, which contribute to two well reported biological activities [5]. Authors have demonstrated the antioxidant and anti-inflammatory potential of leaf extracts, including the inhibition of pro-inflammatory mediators in macrophages stimulated by lipopolysaccharide (LPS) [3], as well as antiangiogenic effects associated with the modulation of oxidative pathways [2]. Additionally, evidence of antitumor activity was observed in liver cell lines (HepG2) [6] and pancreatic (PANC-1) [7], with extracts inducing apoptosis via oxidative stress. Regarding antimicrobial activity, extracts of *T. subulata* inhibit the fungal dimorphism of *Candida albicans* and *Candida tropicalis* [8], and moderate inhibition of bacterial *Streptococcus mutans*, *Staphylococcus aureus*, *Vibrio cholerae*, *Escherichia coli*, *Enterococcus faecalis*, and *Pseudomonas aeruginosa* [7].

The biological effects from *T. subulata* remain unclear in neuronal cell lines, although different authors have reported that the genus Turnera exhibits neuroprotective effects. In this context the antioxidant and anti-inflammatory compounds found in *T. subulata* have been proposed as potential agents to therapeutic treatment of different diseases, principally due to their ability to neutralize reactive oxygen species (ROS), reduce oxidative stress, and affect immune and inflammatory responses, which may help slow disease progressions [4]. However, the neuroprotective potential of *T. subulata* itself has not yet been directly investigated, and its immunomodulatory properties also remain unexplored, despite their relevance in inflammatory regulation and antimicrobial defense.

However, despite these reports, studies investigating the biological activity of *T. subulata* still exhibit limitations in providing an integrated analysis that correlates functional outcomes with the chemical complexity of its extracts. Furthermore, there is a lack of comparative studies evaluating how different extraction solvents influence the efficiency, phytochemical composition, and biological activities of *T. subulata*. In particular, aqueous (AETS) and hydroethanolic (HETS) extracts may differ in their ability to solubilize bioactive compounds due to differences in polarity, which can directly impact their functional properties. In this context, the present study advances current knowledge by providing a comprehensive and integrated assessment of the phytochemical profile (LC-MS/MS) and multiple biological activities of AETS and HETS under standardized experimental conditions.

Therefore, to address thess gaps, the present study aimed to investigate and compare the biological and functional characterization of aqueous and hydroethanolic extracts of *T. subulata*, including cytotoxic, antioxidant, immunomodulatory, and antimicrobial activities. This comparison was designed to elucidate the influence of solvent polarity on the extraction of bioactive compounds and their associated biological effects. Additionally, we realize direct antimicrobial evaluation against drug-resistant Gram-positive and Gram-negative bacteria, which was an opportunity to identify compounds with therapeutic potential in the context of antimicrobial resistance.

To support this integrative approach, biological assays were combined with complementary chemical profiling. Chromatographic analyses coupled with high-resolution mass spectrometry were performed to characterize the chemical composition of the extracts and to identify metabolite classes. The findings presented herein establish a functional–chemical interface that improves the understanding of the biological potential of *T. subulata*. This approach provides a more robust basis for interpreting its pharmacological properties and supports, at least in part, its traditional medicinal use.

## 2. Materials and Methods

### 2.1. Plant Material

*T. subulata* was collected in the city of Esperança (7°02′13″ S, 35°51′33″ W), Brazil. The species identification was confirmed by the Manuel de Arruda Câmera Herbarium of the State University of Paraíba (UEPB). The exsiccata of the species is deposited under the code HACAM00003741.

### 2.2. Preparation of Plant Material and Extracts

For the extracts, the dehydrated aerial parts of *T. subulata* were used. The dried plant material (MV) was processed using a conventional blender for 3 min to ensure a uniform particle size distribution. The MV was characterized in terms of water content, total solids (method 934.06), and total ash (method 940.26) [9].

The dried and processed MV of *T. subulata* was fractionated into 5 g batches. Aqueous extracts were obtained following the method described by Blum-Silva et al. [10], with modifications. Extraction was performed by infusion. For the aqueous extract (AETS), 5 g of processed MV was mixed with 100 mL of distilled water and heated to 85 °C for 20 min in a water bath. The extract obtained was filtered and frozen at −20 °C.

For the hydroethanolic extract (HETS), 5 g of processed MV was mixed with 100 mL of absolute ethyl alcohol solution of PA at a concentration of 40%, heated to 85 °C for 20 min in a water bath, then filtered and subjected to ethanol distillation by rotary evaporation under reduced pressure. The concentrated hydroethanolic extract was frozen at −20 °C.

### 2.3. Determination of Total Solids, Extraction Yield, Phenolic Compounds, Reducing Carbohydrates, and Protein Content

The determination of total solids in the extracts was carried out according to method 934.06 [9]. For analysis, 2.0 mL of extract was measured, then previously evaporated and dried in an oven at 105 °C for 3 h or until constant weight. Subsequently, the containers with the samples were cooled in a desiccator and weighed. The results were expressed as a percentage.

The plant material presented a total solids content of 87.92 ± 2.8%. The total solids content of the extracts was 1.14 ± 0.05% for AETS and 2.40 ± 0.02% for HETS.

The extraction yield was calculated based on the proportion of solids recovered in the extracts relative to the total solids present in the plant material, according to the following Equation (1):

Extraction yield (%) = (total solids in extract/total solids in plant material) × 100(1)

Based on this approach, the extraction yields were 25.93 ± 1.40% for AETS and 54.50 ± 1.79% for HETS.

The content of total phenolic compounds was determined following the Folin–Ciocalteu method [11], using gallic acid to construct the calibration curve (0–0.4 mg/mL). Absorbance readings were measured at 725 nm using a spectrophotometer (SpectraMax i3, San Jose, CA, USA). Results were expressed in mg gallic acid equivalent (GAE)/100 mL. Reducing carbohydrate content was determined using the colorimetric method using 3,5-dinitrosalicylic acid (DNS), as described by Miller [12]. The calibration curve was constructed using glucose (0–4%). Absorbance readings were measured at 575 nm using a spectrophotometer (SpectraMax i3, San Jose, CA, USA). Results were expressed in mg of glucose/100 mL of sample. Total protein content was determined using the Lowry method. [13]. Absorbance was measured at 750 nm using a spectrophotometer (SpectraMax i3, San Jose, CA, USA). The calibration standard curve was prepared with bovine serum albumin (BSA) solutions with concentrations between 0 and 2000 µg/mL. Results were expressed in mg of BSA/100 mL.

### 2.4. Chemical Profile by LC-MS

The previously frozen extracts were lyophilized for 48 h, packaged in polypropylene tubes protected from light and moisture, and stored at 25 ± 2.5 °C until analysis. The samples were reconstituted to a final concentration of 200 µg/mL and analyzed using a liquid chromatography–mass spectrometry (LC-MS) system composed of ultra-high-performance liquid chromatography (UHPLC) (LC-40D X3) coupled to a quadrupole time-of-flight (QTOF) mass spectrometer (LCMS-9050, Shimadzu, Kyoto, Kyoto Prefecture, Japan) with an electrospray ionization (ESI) source. Chromatographic separation was performed on a C18 column (150 × 4.6 mm, 2.7 µm), using a mobile phase consisting of Milli-Q^®^ (Merck Millipore, Burlington, MA, USA) water (A) and methanol (B), under a linear gradient (95–0% A) for 27 min, a flow rate of 0.3 mL/min, and a temperature of 40 °C. The analyses were conducted in negative ionization mode, with a capillary voltage of −3.0 kV or 4.0 kV, a nebulizer gas flow rate of 3.0 L/min, a drying gas flow rate of 10 mL/min, and an interface temperature of 300 °C. Data acquisition was performed in data-independent acquisition (DIA) mode, in the m/z range of 100–1200 for precursor ions and 50–1200 for product ions, with an event time of 0.034 s, a collision energy of 30 eV, and argon at 230 kPa. The data were processed using MS-DIAL (Mass Spectrometry Data-Independent Analysis) software (v.5.5) for compound annotation based on public spectral libraries, and chromatographic visualization was performed using MZmine (Mass Spectrometry Data Analysis Platform) (v.4.8.3).

### 2.5. Cellular Viability

For cell viability assays, SH-SY5Y cells were cultured in DMEM F12 medium supplemented with 10% fetal bovine. Cells were allowed to adhere and grow until they reached 70–80% confluence before treatments and then seeded in 96-well plates at a density of 1 × 10^5^ cells/mL.

#### 2.5.1. MTT Reduction Assay

The MTT (3-[4,5-dimethylthiazol-2-yl]-2,5-diphenyl-tetrazolium) reduction assay was used to quantify the viability of SH-SY5Y cells when treated with different concentrations of *T. subulata* extracts [14]. For the assay, the culture medium of SH-SY5Y cells was aspirated, and culture medium plus the necessary volume of extract was added to obtain a curve with concentrations of 5000, 500, 50, 5, 0.5, and 0.05 µg/mL. The plate was incubated for 24 h at 37 °C in an incubator with 5% CO_2_. Subsequently, the medium was aspirated and the cells washed with MTT solution (1 mg/mL), and the plate was incubated at 37 °C for 2 h with 5% CO_2_. After 2 h, the MTT solution was aspirated, and 100 µL of isopropanol, used to dissolve formazan crystals, was added to each well of the plate. Absorbance readings were performed at 560 nm and 630 nm using a spectrophotometer (SpectraMax i3, San Jose, CA, USA).

#### 2.5.2. Sulforodamine (SRB) Assay and Incorporation

The sulforhodamine incorporation assay (SRB) is based on the binding of the SRB dye to total cellular protein [15]. For this assay, SH-SY5Y cells were cultured and treated with different concentrations of the extracts as described for the MTT assay. After an incubation period at 37 °C for 2 h in 5% CO_2_, the culture medium was aspirated and the cells were fixed with 200 µL of trichloroacetic acid (TCA 10%) and incubated at 4 °C for 1 h. The wells of the plate containing the cells were subsequently washed with cold water, and the excess water was decanted and dried. In each well with cells, 100 µL of SRB dye (0.4% SRB in 1% acetic acid) was added and incubated for 60 min. The cells were then washed with 1% acetic acid four times. Then, 200 µL of 10 mM TRIS was added to dilute the dye. The plates were shaken and absorbance read at 510 nm and 620 nm using a spectrophotometer (SpectraMax i3, San Jose, CA, USA). Cell survival was expressed as the difference in absorbance obtained with respect to the control.

### 2.6. Hydroxyl Radical-Mediated 2-Deoxyribose Oxidation Assay

The quantification of the hydroxyl radical from the Fenton reaction was performed using the oxidative degradation assay of 2-deoxyribose (2-DR). For this, 2-DR was incubated with a hydroxyl radical-generating system (induced control: 100 µL of 2-DR + 100 µL of ferrous sulfate + 100 µL of H_2_O_2_ + 700 µL of phosphate buffer), where 2-DR is degraded to malondialdehyde (MDA), which condenses with thiobarbituric acid (TBA) to form a pink chromophore [16]. Samples diluted to achieve concentrations of 5000, 500, 50, 5, 0.5, and 0.05 µg/mL were added to the system (induced control). Trolox, obtained from Sigma-Aldrich with HPLC grade, was used as a standard antioxidant. Trolox was added to the system (induced control). Readings were performed on a spectrophotometer (SpectraMax i3, San Jose, USA) at 532 nm. The results were expressed as % hydroxyl-induced 2-deoxy-D-ribose oxidation.

### 2.7. Total Antioxidant Activity (TRAP) Assay

The TRAP was determined by measuring the luminescence inhibition capacity generated by the AAPH-induced reaction with luminol, being proportional to the antioxidant potential of the extracts evaluated at concentrations of 5000, 500, 50, 5, 0.5, and 0.05 µg/mL, according to the method described by Dresch et al. [17], with adaptations. The luminescence inhibition capacity was measured by adding AAPH (10 mM) to glycine buffer (100 mM, pH 8.6) in a light-protected flask with a capacity of 20 mL. Then, 62.5 µL of luminol (4 mM) was added to the AAPH solution and allowed to stabilize for 2 h. After the stabilization time, 20 µL of 40 µM Trolox solution or 20 µL of the sample was added, and luminescence was measured using a Varioskan™ LUX instrument (Thermo Fisher Scientific Inc., Waltham, MA, USA), with readings taken every 5 min for 2 h at 452 nm and 489 nm. AUC was calculated using GraphPad Prism 8.0 software.

### 2.8. Capacity to Scavenge Free Radicals (DPPH and ABTS)

To evaluate the ability of the extracts to scavenge DPPH (2,2-Diphenyl-1-picrylhydrazyl) and ABTS (2,2-azino-bis(3-ethylbenzothiazoline-6-sulfonic acid)) radicals, a concentration range different from that applied in the cell viability tests (MTT and SRB), hydroxyl radical scavenging activity, and TRAP (0.05–5000 µg/mL) were evaluated due to the fundamental differences between the assays. Assays such as DPPH and ABTS are more sensitive and have a limited dynamic and linear range; very high extract concentrations tend to saturate the signal or introduce optical interference (turbidity/background absorbance). Furthermore, maintaining a low percentage of vehicles in the assay imposes practical limits on the concentration tested. The chosen concentration range (15.62–1000 µg/mL) provides adequate resolution for estimating the inhibition capacity of DPPH and ABTS radicals [18,19].

For the DPPH assay, 40 µL of the sample was mixed with 250 µL of DPPH (1 mM in methanol) and left to stand for 25 min at room temperature and protected from light. Ascorbic acid (≥99% purity, Sigma-Aldrich) was used as a standard at 5–100 µg/mL, and methanol PA (≥99.8% purity) was used as the blank. Absorbance readings were taken at 517 nm using a microplate reader (BioTek UQuant MQX200, Winooski, VT, USA) [20].

For the ABTS assay, the radical was obtained by mixing 5 mL of 7 mM ABTS solution with 88 µL of 140 mM potassium persulfate solution. This mixture remained in the dark at room temperature for 16 h before use. The ABTS radical solution was diluted with ethanol until an absorbance of 0.7 nm was obtained at 734 nm. A measurement of 10 µL of the sample was mixed with 1 mL of the ABTS radical and allowed to react for 6 min. Absorbance readings were performed using Spectrophotometer Shimadzu UV-1800 (Kyoto, Japan) at 734 nm. Ascorbic acid (≥99% purity, Sigma-Aldrich) was used as a standard at 5–100 µg/mL [21].

The inhibition capacity (%) was calculated according to Equation (2):

DPPH/ABTS inhibition (%) = [(Ac − As)/(Ac)] × 100(2)

where Ac is the absorbance of the blank and As is the absorbance of the sample.

The IC_50_ values were determined by nonlinear regression using a four-parameter logistic model, which better describes the sigmoidal behavior of dose–response curves observed in DPPH and ABTS assays.

### 2.9. Immunomodulatory Activity

#### 2.9.1. Cytotoxic Activity in Murine Splenocytes

For immunomodulatory activity, only the hydroethanolic extract of *T. subulata* was used, selected for presenting the highest antioxidant response among the extracts previously analyzed. Splenocytes obtained from female BALB/c mice (6–8 weeks old) were used. All procedures were conducted after approval by the Ethics Committee on the Use of Animals of the Federal University of Pernambuco (UFPE) (authorization no. 055/2016). The animals were anesthetized and euthanized for aseptic removal of the spleen, from which splenocytes were isolated according to the methodology described by Lessa et al. [22]. Only cell cultures with viability greater than 98%, determined by the trypan blue exclusion method, were used in subsequent assays.

Given that the immunomodulation assay requires that the concentrations used do not compromise cell viability, it was decided to study a range of concentrations below the cytotoxicity threshold observed in the preliminary results of the MTT and SRB assays (i.e., ≤50 µg/mL), starting from serial dilutions that preserved cell viability and allowed observation of functional modulation [22,23]. Thus, for the assay, splenocytes were cultured with RPMI 1640 medium (Sigma-Aldrich, St. Louis, MO, USA) containing 10% fetal bovine serum in an incubator at 37 °C with 5% CO_2_. The cells were treated with different concentrations of the hydroethanolic extract (3, 125, 6, 250, 12.5, 25, and 50 µg/mL) for 24 h under the same incubation conditions. Untreated (control) cells and cells treated with the extract were collected by centrifugation (450× *g*, 4 °C, 10 min) and washed twice with phosphate-buffered saline (PBS). The cytotoxicity of the extract on splenocytes was evaluated by flow cytometry using the FTIC Annexin V Apoptosis Detection Kit II (BD Biosciences, Franklin Lakes, NJ, USA), according to the manufacturer’s instructions. Cells stained with Annexin V-FITC were considered apoptotic, while those positive for propidium iodide (PI) were classified as necrotic. Double-negative cells were considered viable.

Data acquisition was performed using a FACSCalibur flow cytometer (BD Biosciences) with a collection of 10,000 events per sample. Analyses were conducted using CellQuest Pro software (version 5.2.1). All experiments were performed in quintuplicate, in two independent assays.

#### 2.9.2. Cytokine Release

For cytokine release analysis, a concentration of 12.5 µg/mL was selected as an intermediate dilution within the tested range (3.12–50 µg/mL) that preserved cell viability (>90%) as determined by Annexin V/P analysis, ensuring that any changes in cytokine levels reflect functional modulation and not cytotoxic effects. Furthermore, a moderate concentration was chosen to minimize potential matrix interferences in the Cytometric Bead Array analysis (detection range 2–5000 pg/mL).

Cytokine quantification was performed using a Cytometric Bead Array Mouse Th1/Th2/Th17 cytokine kit (BD Biosciences, Franklin Lakes, NJ, USA), employing supernatants from splenocyte cultures incubated in the absence (control) and presence of 12.5 µg/mL of the hydroethanolic extract. The levels of interleukins IL-4, IL-10, IL-12, and IL-17 were determined according to the manufacturer’s instructions. Data acquisition and analysis were conducted using FCAP Array software (version 3.1). Standard curves for each cytokine (IL-4, IL-10, IL-12, and IL-17) were constructed in the range of 0–5000 pg/mL, with a detection range of 2–5000 pg/mL.

### 2.10. Antimicrobial Activity Assay

For the antimicrobial assays, standard strains from the American Type Culture Collection (ATCC) were used, including two Gram-positive bacteria (*Staphylococcus aureus* ATCC 25923 (MSSA) and *Staphylococcus aureus* ATCC 6538) and two Gram-negative bacteria (*Escherichia coli* ATCC 8739 and *Escherichia coli* ATCC 25922). All bacterial strains were maintained at −70 °C in BHI broth supplemented with 20% glycerol and replicated on BHI agar plates (KASVI, Curitiba, PR, Brazil) before the assays.

#### 2.10.1. Minimum Inhibitory Concentration (MIC)

To determine the MIC, the Brain Heart Infusion (BHI) broth microdilution method was used, based on protocols M07 [24] and M100 [25], with modifications. For inoculum preparation, colonies from BHI agar plates were suspended in sterile 0.9% NaCl solution (VETEC Química, Rio de Janeiro, Brazil) to obtain turbidity corresponding to McFarland standard 0.5 (1 × 10^8^ CFU/mL) [25]. The MIC was defined as the lowest concentration of the extract that produced 100% inhibition of visible bacterial growth compared to the negative controls (BHI broth) and positive controls (BHI broth plus bacteria). The bacterial species were *Staphylococcus aureus* ATCC 25923 (MSSA), *Staphylococcus aureus* ATCC 6538, *Escherichia coli* ATCC 25922 and *Escherichia coli* ATCC 8739. For the bacterial species in cases where visual determination of inhibition was difficult or ambiguous, the analysis was supplemented by the XTT (2,3-bis-(2-methoxy-4-nitro-5-sulfophenyl)-2H-tetrazolium-5-carboxanilide) reduction assay, as described by Tunney et al. [26], with modifications. A measurement of 50 µL of aqueous XTT solution (1 mg/mL) containing menadione (152 µM) was added to each well after 24 h of incubation and incubated again for 1 h at 35 ± 2 °C. The absorbance of the reduced XTT was measured at 492 nm using a microplate reader (BioTek Synergy HT, Winooski, VT, USA). All tests were performed in triplicate, and the results were expressed as the percentage of growth inhibition, calculated by the difference between bacterial growth in the presence of the extract and growth in the positive control (BHI broth plus bacteria). The same assay was also performed with the reference antibiotics (vancomycin and colistin), both acquired from Sigma-Aldrich.

For this assay, the series of concentrations of the hydroethanolic extract (5.00; 2.50; 1.25; 0.625; 0.3125; 0.1562; 0.078; 0.039 mg/mL) was defined based on established practices for inhibitory activity testing of crude extracts. MIC assays with plant extracts frequently employ maximum concentrations in the mg/mL range, as the active principles may be present in low proportions in the total extract, and the reported MIC values for crude materials vary widely [27].

#### 2.10.2. Synergism Trial

The study of potential synergism between the hydroethanolic extract of *T. subulata* and the antibiotic vancomycin was conducted following the methodology described by Jeong et al. [28], with modifications. *S. aureus* ATCC 25923 MSSA and *S. aureus* ATCC 6538 were selected for this assay based on their previously observed susceptibility profiles in the MIC test, which demonstrated a more pronounced inhibitory response to the extract. These strains were inoculated at a concentration of 10^4^ CFU in BHI broth. 96-well microplates were used. Wells in horizontal rows were treated with serial dilutions of the extract (5–0.078 mg/mL), and wells in vertical columns were treated with serial dilutions of vancomycin (0.0078–8 µg/mL). Antibiotic concentrations were selected according to the established cutoff points for vancomycin (resistant > 2 µg/mL; sensitive < 2 µg/mL) [25]. After preparation, the plates were incubated for 24 h at 35 ± 2 °C. Bacterial growth was determined by evaluating turbidity and confirmed by adding 50 µL of XTT solution, followed by a 1 h incubation and absorbance reading at 492 nm using a microplate reader (BioTek Synergy HT, Winooski, VT, USA). All tests were performed in duplicate.

### 2.11. Statistical Analysis

Descriptive and inferential statistics were used to analyze the data obtained from biological assays and chemical characterization. Results are expressed as mean ± standard deviation. Physicochemical and biochemical characterization data were analyzed using one-way analysis of variance (ANOVA) followed by Sidak’s post hoc test. Cytotoxicity and antioxidant assays (excluding DPPH and ABTS) were analyzed using two-way ANOVA, followed by Dunnett’s post hoc test for comparisons with the control and Sidak’s test for comparisons between extracts. The DPPH and ABTS assay results were analyzed descriptively, without inferential statistical testing. Immunomodulatory and antimicrobial activity data were analyzed using one-way ANOVA followed by Dunnett’s post hoc test in comparison to the control. Statistical significance was set at *p* < 0.05. All analyses and graphs were performed using GraphPad Prism version 8.0 and Microsoft Excel^®^ 2010.

## 3. Results

### 3.1. Physicochemical and Biochemical Composition of Plant Material and Extracts of T. subulata

The plant material (MV) of *T. subulata* used in the preparation of the extracts presented a moisture content of 12.08 ± 2.81%, indicating low water retention. Consequently, the percentage of total solids was 87.91 ± 2.81%, reflecting a high concentration of dry matter available for extraction. Furthermore, the ash content of 4.99 ± 0.06% evidenced the presence of a considerable mineral fraction.

Table 1 shows the values for total solids, reducing carbohydrates, proteins, total phenolic compounds, and extraction yield for the aqueous (AETS) and hydroethanolic (HETS) extracts. HETS showed significantly higher values (*p* < 0.0001) compared to AETS for total solids, protein, total phenolic compounds, and yield. For carbohydrate reduction, AETS showed significantly higher values than HETS (*p* < 0.0001), demonstrating a significant influence of the solvent on the extraction of various compounds.

### 3.2. Chemical Profile of T. subulata

The LC–MS/MS analysis of *T. subulata* extracts allowed the putative annotation of eleven compounds (Table 2). The profile included organic acids, phenolic acids, and mainly flavonoids. Early eluting compounds (tR < 6 min) corresponded to primary metabolites, such as gluconic acid and citric acid. Compounds detected at intermediate retention times (8–11 min) were putatively annotated as phenolic acids, including gentisic acid -O--hexoside, feruloylquinic acid, and O-caffeoylquinic acid. At higher retention times (tR > 13 min), the chromatogram was dominated by flavonoids, especially glycosylated derivatives of apigenin, such as homoorientin, vitexin, vitexin-O-hexoside I, vitexin-O-hexoside II apiin, and apigenin-O-hexoside. All compounds were tentatively putatively annotated based on accurate mass and MS/MS fragmentation, using GNPS2 and MS/MS Public Library databases.

**Figure 1:**
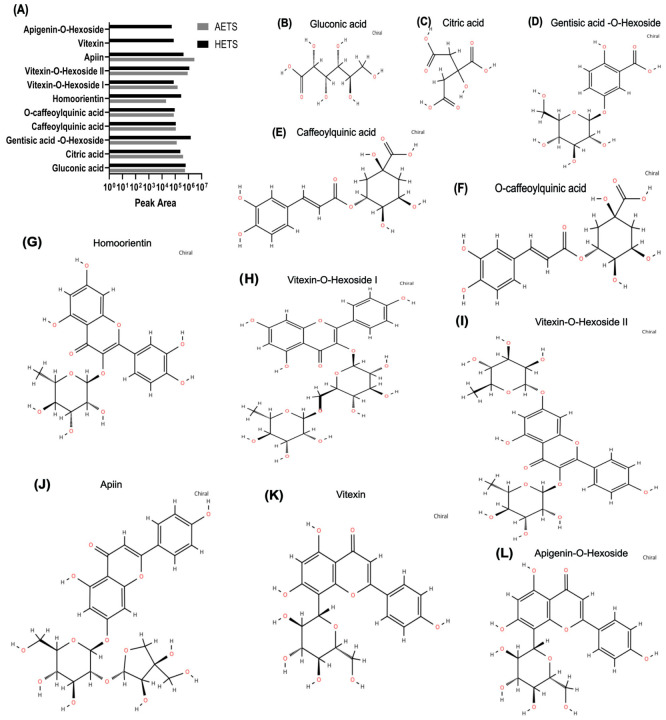
Relative abundance and proposed chemical structures of compounds putatively annotated in *T. subulata* extracts. (**A**) Bar graph showing the relative abundance of annotated metabolites in aqueous (AETS) and hydroethanol (HETS) extracts. (**B**–**L**) Proposed chemical structures of the identified compounds, including organic acids (gluconic acid and citric acid), phenolic acids, and flavonoid glycosides such as homoorientin, vitexin, vitexin-O-hexoside I, vitexin-O-hexoside II, apiin, and apigenin-O-hexoside. The chemical structures were obtained from the PubChem database (https://pubchem.ncbi.nlm.nih.gov/) (accessed on 1 May 2026).

Figure 1 presents the relative abundance and proposed chemical structures for the presumably identified compounds. The bar graph (Figure 1A) shows the differences between the AETS and HETS extracts, with a higher relative abundance of flavonoid glycosides, particularly vitexin and apiin derivatives, in HETS. Panels B–L represent the proposed structures for the identified metabolites, highlighting the chemical diversity of the extracts, which include organic acids (such as gluconic and citric acids), phenolic acids, and flavonoid glycosides. The identified compounds exhibit multiple hydroxyl groups and glycosidic moieties, which is consistent with their polar nature and retention behavior in liquid chromatography [40]. Furthermore, several structures are indicated as chiral, suggesting the presence of stereogenic centers [41].

### 3.3. MTT and SRB Cytotoxicity

**Figure 2:**
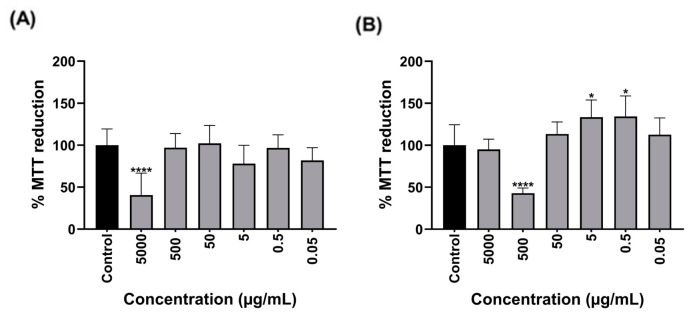
Evaluation of MTT reduction in SH-SY5Y cells treated with increasing concentrations (0.05–5000 µg/mL) of AETS (**A**) and HETS (**B**) extracts. The control consisted only of SH-SY5Y cells with culture medium. The values presented are mean ± standard deviation, with n = 6. Statistical analysis was performed using two-way ANOVA with Dunnett’s multiple comparisons in relation to the control and Sidak’s test for comparison between extracts. Significance values were represented as * *p* < 0.05 and **** *p* < 0.0001.

The solvents used to obtain the *T. subulata* extracts significantly influenced cell viability in the MTT assay (Figure 2). Statistically significant differences were observed between the AETS and HETS extracts at all concentrations, except at the 50 µg/mL concentration. In the AETS extract, only the highest concentration tested (5000 µg/mL) significantly reduced the viability of SH-SY5Y cells, resulting in a viability of 40.53% compared to the control. For the HETS extract, a cytotoxic effect was observed at 50 µg/mL, with a significant reduction in cell viability (42.85%) compared to the control. At the highest HETS concentration (5000 µg/mL), the percentage of MTT reduction did not differ significantly from the control group. Although serial dilutions were performed to minimize potential interference, this non-monotonic response may still be associated with effects of the extract on the assay readout, possibly due to its intrinsic color components. In addition, alterations in cellular metabolic activity at higher concentrations cannot be excluded.

**Figure 3:**
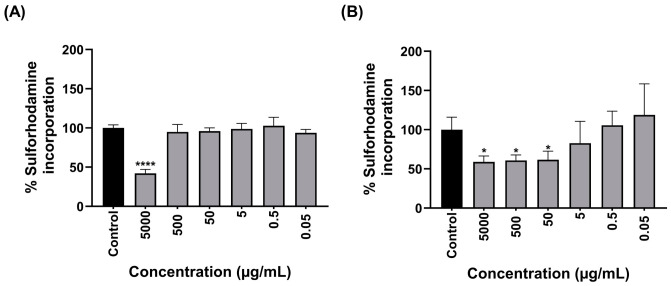
SRB incorporation in SH-SY5Y cells treated with increasing concentrations (0.05–5000 µg/mL) of AETS (**A**) and HETS (**B**) extracts. The control consisted only of SH-SY5Y cells with culture medium. The values presented are mean ± standard deviation, with n = 6. Statistical analysis was performed using two-way ANOVA with Dunnett’s multiple comparisons in relation to the control and Sidak’s test for comparison between extracts. Significance values were represented as * *p* < 0.05 and **** *p* < 0.0001.

The cytotoxicity of the AETS and HETS extracts was evaluated by the SRB assay in SH-SY5Y cells (Figure 3). The SRB assay was considered a complementary protein-based endpoint to support the MTT results. The results showed a statistically significant difference between the two extracts at concentrations of 500 and 50 µg/mL (*p* < 0.01). The AETS extract maintained cell viability above 90% in the range of 0.05 to 500 µg/mL, indicating low cytotoxicity under these conditions. However, at the highest concentration (5000 µg/mL), AETS significantly reduced cell viability (*p* < 0.0001), reaching 42.10%. The HETS extract showed lower cell viability than that observed for AETS at equivalent concentrations, ranging from 60.72% to 118.7% in the range of 500 to 0.05 µg/mL (Figure 3B). Compared to the control, concentrations of 5000, 500, and 50 µg/mL of the HETS extract significantly reduced cell viability (*p* < 0.05).

### 3.4. Effect of Extracts on Hydroxyl Radical-Mediated Oxidation

**Figure 4:**
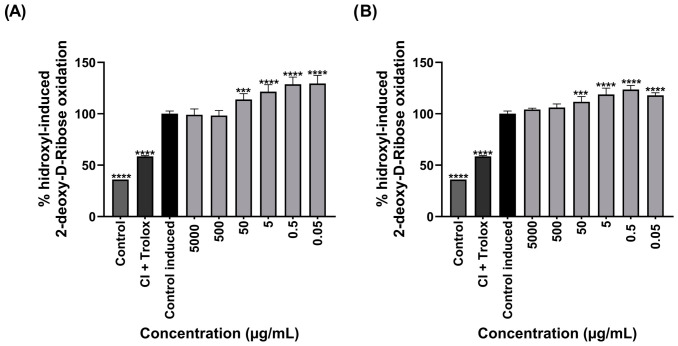
Effect of AETS and HETS extracts on hydroxyl radical-mediated oxidation of 2-deoxy-D-ribose (2-DR). Increasing concentrations (0.05–5000 µg/mL) of the AETS (**A**) and HETS (**B**) extracts were analyzed. The induced control is the production of MDA from the oxidation of 2-DR with FeSO_4_ and H_2_O_2_ alone. Another control group consists of the system without the generation of hydroxyl radicals. The CI + Trolox consists of the induced control (MDA production from the oxidation of 2-DR with FeSO_4_ and H_2_O_2_) with the addition of 200 nM Trolox as a standard antioxidant. The values presented are the mean ± standard deviation with n = 3. Statistical analysis was performed using Dunnett’s two-way ANOVA with multiple comparisons in relation to the induced control and Sidak’s test for comparison between extracts. Significance values were represented as *** *p* < 0.001 and **** *p* < 0.0001.

Extracts of *T. subulata* did not exert an inhibitory effect on the hydroxyl radical-mediated oxidation of 2-deoxy-D-ribose (Figure 4). Instead, a dose-dependent increase in 2-DR degradation was observed compared to the induced control, with no extract recapitulating the suppression of oxidation observed when Trolox was added to the induced control. This increase in hydroxyl radical-mediated oxidation of 2-DR was significant even at a concentration of 50 µg/mL (*p* < 0.001), for both the AETS extract (Figure 4A) and the HETS extract (Figure 4B). The solvent used in the preparation of the extracts was not significantly influential in this result, except at a concentration of 0.05 µg/mL, where a statistically significant difference with *p* < 0.05 was observed between the aqueous (AETS) and hydroethanolic (HETS) extracts.

### 3.5. Total Antioxidant Activity (TRAP)

**Figure 5:**
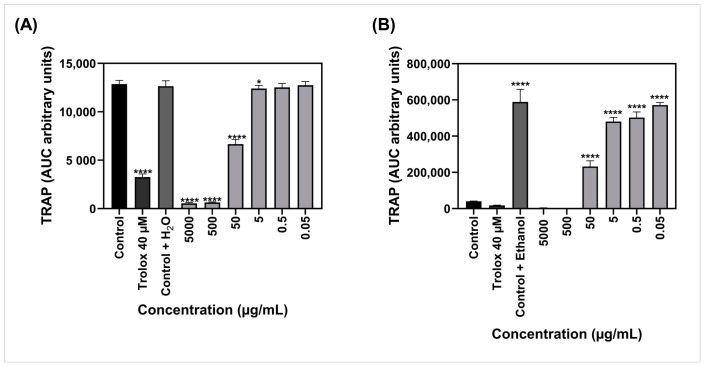
Antioxidant activity of increasing concentrations (0.05–5000 µg/mL) of AETS (**A**) and HETS (**B**) extracts investigated by TRAP. Oxidation was triggered by the presence of the AAPH free radical, both in the absence of an antioxidant agent (induced control and induced control + H_2_O) and in the presence of Trolox at a concentration of 40 µM. The values presented are the mean ± standard deviation with n = 3. Statistical analysis was performed using two-way ANOVA with Dunnett’s multiple comparisons in relation to the control and Sidak’s test for comparison between extracts. Significance values were represented as * *p* < 0.05 and **** *p* < 0.0001.

Figure 5 shows the area under the curve (AUC) values obtained at different concentrations of *T. subulata* extracts for total antioxidant activity (TRAP). The solvents used to prepare the extracts showed a significant influence (*p* < 0.0001) for concentrations of 50, 5, 0.5, and 0.05 µg/mL and for the added solvent control (*p* < 0.0001). The AETS extract (Figure 5A) showed significant antioxidant activity starting at a concentration of 50 µg/mL (*p* < 0.0001). However, for the HETS extract, despite a similar downward trend, at equivalent concentrations observed for AETS, this antioxidant activity was not statistically significant (*p* > 0.05). In the HETS extract (Figure 5B), a pro-oxidant effect was observed at concentrations below 50 µg/mL, suggesting a dose-dependent antioxidant profile with specific characteristics of the solvent used in the extraction. Furthermore, the solvent tested in the control, in Figure 4B, ethanol at a concentration of 40%, the concentration used to obtain the extract, caused an increase in AUC, indicating a possible effect of the solvent on the antioxidant activity results investigated by TRAP. However, we emphasize that in the HETS extracts, the ethanol was evaporated before the extract was applied in the assays.

### 3.6. Antioxidant Potential by DPPH and ABTS

**Figure 6:**
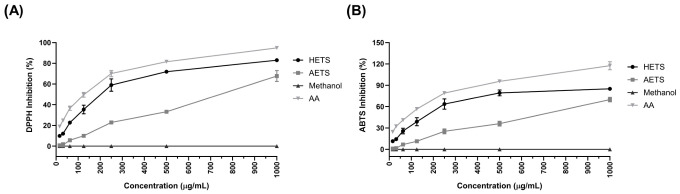
Percentage of DPPH (**A**) and ABTS (**B**) radical scavenging for *T. subulata* extracts at increasing concentrations (15.62–1000 µg/mL). Ascorbic acid was used as a standard and methanol as a solvent. The values presented are the mean ± standard deviation with n = 3. The IC_50_ values were determined by nonlinear regression using a four-parameter logistic model, yielding 196.5 µg/mL (DPPH) and 172.8 µg/mL (ABTS) for the hydroethanolic extract (HETS), and 728.4 µg/mL (DPPH) and 689.7 µg/mL (ABTS) for the aqueous extract (AETS). Data are presented descriptively without inferential statistical analysis.

To complement the study of the antioxidant activity of the extracts, DPPH and ABTS radical scavenging assays (Figure 6) were used in a specific concentration range (15.62–1000 µg/mL), due to the sensitivity of the assays. An increase in the percentage of DPPH (Figure 6A) and ABTS (Figure 6B) radical scavenging was observed, depending on the concentration of the AETS and HETS extracts. At the highest evaluated concentration, the HETS extract (maximum of 83.05 ± 0.05% for DPPH and 85.1 ± 1.5% for ABTS) showed significantly higher values compared to AETS (maximum of 67.75 ± 5.26% for DPPH and 69.9 ± 3.16% for ABTS) with *p* < 0.05.

### 3.7. Cytotoxicity in Splenocytes

The cytotoxicity assessment of the HETS extract on splenocytes was performed as an initial step to define safe concentrations to be used in functional immunomodulation assays. Only the HETS extract was evaluated due to the results observed in antioxidant activity tests, especially the DPPH and ABTS radical scavenging assays, which demonstrated greater antioxidant activity compared to the AETS extract. Therefore, the AETS extract was not included in this assay.

**Figure 7:**
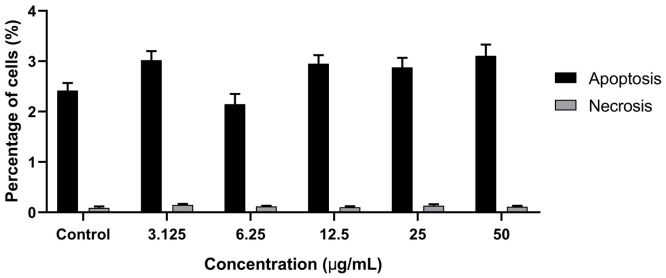
Evaluation of the cytotoxicity of the hydroethanolic extract (HETS) of *T. subulata* on splenocytes of BALB/c mice by flow cytometry. Cells were treated with different concentrations of the extract (3.125–50 µg/mL). The values presented are the mean ± standard deviation with n = 5.

Splenocytes exhibited viability greater than 98% before treatment, ensuring the reliability of the assays. Cells were exposed to increasing concentrations of the extract (3.125–50 µg/mL), selected because they were below the cytotoxicity threshold observed in the MTT (Figure 2) and SRB (Figure 3) assays. Figure 7 shows the percentage of cells undergoing apoptosis and necrosis after treatment with the HETS extract.

The HETS extract did not induce apoptosis or necrosis rates at any of the concentrations evaluated. The percentage of apoptotic cells ranged from 2.15 ± 0.12% to 3.11 ± 0.20%, while necrosis remained below 0.15 ± 0.03% at all concentrations, with values comparable to the control group (apoptosis: 2.42 ± 0.10%; necrosis: 0.09 ± 0.01%). Therefore, the HETS extract demonstrated a non-cytotoxic profile even at the highest concentration tested (50 µg/mL).

### 3.8. Expression of Anti-Inflammatory and Pro-Inflammatory Cytokines

**Figure 8:**
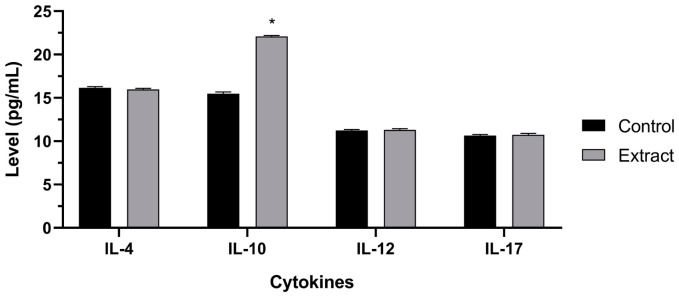
Cytokine profile in splenocytes treated with 12.5 µg/mL of HETS extract. Data are presented as mean ± standard deviation of two independent experiments, with n = 5. Statistical analysis was performed using one-way ANOVA with Dunnett’s test compared to the control. Significance values were represented as * *p* < 0.05.

The cytokine profile generated by splenocyte cells was significantly altered (*p* < 0.05) after treatment with the HETS extract at a concentration of 12.5 µg/mL. The concentration of 12.5 µg/mL was selected because it is an intermediate dilution within the tested range (3.12–50 µg/mL) that preserved cell viability (>90%) as per the apoptosis assay. The values observed in Figure 8 show that the levels of IL-10 increased significantly (*p* < 0.05), while no significant change was observed in the levels of IL-4, IL-12, or IL-17. As the cytokine analysis was conducted at a single concentration, these results should be interpreted as preliminary evidence of the immunomodulatory potential of the extract.

### 3.9. Antimicrobial Activity

#### 3.9.1. Minimum Inhibitory Concentration (MIC) of *T. subulata* Extract

**Figure 9:**
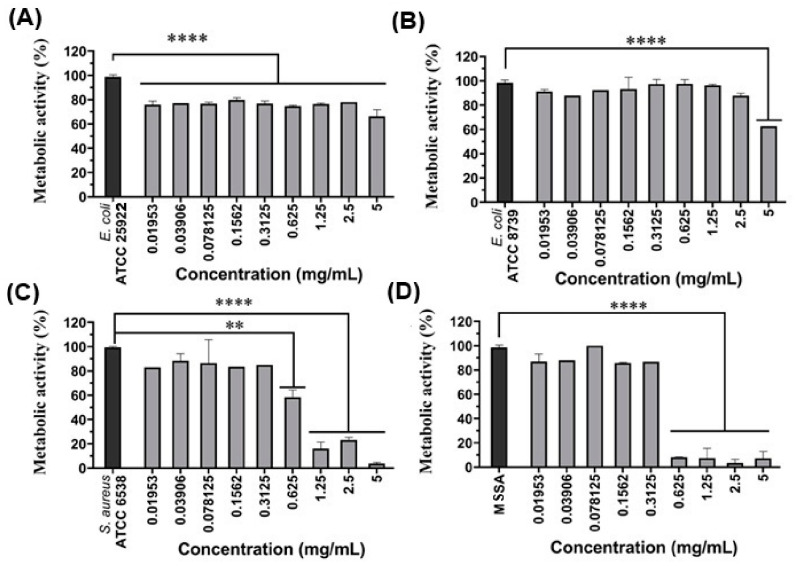
Minimum inhibitory concentration of HETS extract at different concentrations (0.01953–5 mg/mL) against *E. coli* ATCC 25922 (**A**), *E. coli* ATCC 8739 (**B**), *S. aureus* ATCC 6538 (**C**) and *S. aureus* ATCC 25923-MSSA (**D**). For the bacterial species in cases where visual determination of inhibition was difficult or ambiguous, the analysis was supplemented by the XTT reduction assay. Results are presented as mean ± standard deviation with n = 3. Statistical analysis was performed using one-way ANOVA with Dunnett’s test, comparing to the control (culture medium plus bacteria). Significance values were represented as ** *p* < 0.01 and **** *p* < 0.0001.

The AETS extract was not evaluated in the antimicrobial assays, and only the HETS extract was tested. Metabolic evaluation of *E. coli* ATCC 25922 after treatment with the HETS extract showed that the extract did not promote relevant inhibition for all concentrations tested (0.01952–5 mg/mL) (Figure 9A). Although statistically different (*p* < 0.00001), the absolute reduction in metabolic activity was modest, remaining between approximately 66.32% and 79.82%. Even at the highest concentration (5 mg/mL), cellular activity remained elevated, indicating the absence of a significant inhibitory effect. Thus, it was not possible to determine a MIC within the evaluated range for *E. coli* ATCC 25922.

For *E. coli* ATCC 8739 (Figure 9B), a similar pattern was observed. Most concentrations maintained metabolic activity above 80 reduction only at the 5 mg/mL dose, where bacterial metabolism dropped to approximately 62.63%. Despite statistical significance (*p* < 0.0001), the HETS extract did not reach levels that characterize robust inhibition of the growth of this bacterium. Similar to the ATCC 25922 strain, it was not possible to estimate a MIC within the tested range.

Unlike Gram-negative strains, *S. aureus* ATCC 6538 demonstrated greater sensitivity to the HETS extract (Figure 9C). The lowest concentrations (0.01953–0.625 mg/mL) showed metabolic activity above 70%, although with a significant difference from the control (*p* < 0.01). From a concentration of 1.25 mg/mL, a sharp decrease was observed, with 16.19% activity, and even greater reductions at concentrations of 5 mg/mL (3.74% of metabolic activity). These results indicate an approximate MIC of 1.25 mg/mL, with growth inhibition at concentrations ≥ 2.5 mg/mL.

The *S. aureus* MSSA strain showed the most sensitive profile to the HETS extract, with strong inhibitory activity (Figure 9D). Lower concentrations maintained metabolic activity above 80%; however, a significant reduction was observed even at a concentration of 0.625 mg/mL, with metabolic activity of 8.18%. These values suggest an MIC close to 0.625 mg/mL.

#### 3.9.2. Minimum Inhibitory Concentration (MIC) of Antibiotics

**Figure 10:**
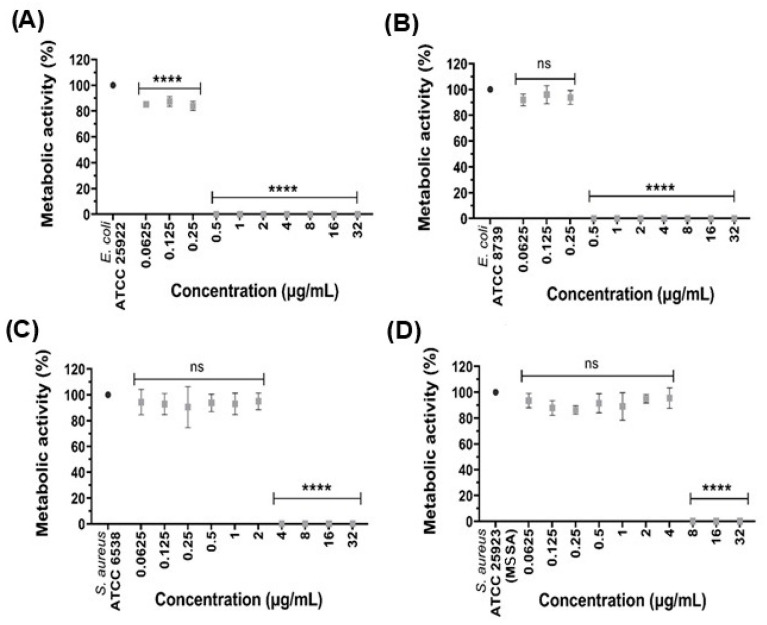
Minimum inhibitory concentration of the antibiotic colistin against *E. coli* ATCC 25922 (**A**) and *E. coli* ATCC 8739 (**B**), and vancomycin against *S. aureus* ATCC 6538 (**C**) and *S. aureus* ATCC 25923-MSSA (**D**). For the bacterial species in cases where visual determination of inhibition was difficult or ambiguous, the analysis was supplemented by the XTT reduction assay. Results are presented as mean ± standard deviation with n = 3. Statistical analysis was performed using one-way ANOVA with Dunnett’s test, comparing to the control (culture medium plus bacteria). Significance values were represented as **** *p* < 0.0001.

The bacterial strains used to test the MIC of the HETS extract were also evaluated against the antibiotic colistin, indicated as a standard for *E. coli* strains, and vancomycin, indicated as a standard for *S. aureus* strains. The antibiotic concentrations ranged from 0.0625 µg/mL to 32 µg/mL, following the method applied for determining the MIC of the HETS extract (Figure 10).

Colistin showed a significant reduction (*p* < 0.001) in the metabolic activity of *E. coli* ATCC 25922 even at lower concentrations (0.0625–0.25 µg/mL); however, with still high values (>80%) (Figure 10A). Bacterial growth inhibition by colistin was observed from a concentration of 0.5 µg/mL, where complete extinction of the metabolic activity of *E. coli* ATCC 25922 was observed. The *E. coli* ATCC 8739 strain showed similar behavior, with complete extinction of metabolic activity from a concentration of 0.5 µg/mL (Figure 10B).

For the *S. aureus* ATCC 6538 strain (Figure 10C), vancomycin showed considerable inhibition of bacterial growth starting at a concentration of 4 µg/mL. However, for the *S. aureus* ATCC 25923-MSSA strain, growth inhibition was only observed starting at a concentration of 8 µg/mL, showing greater resistance of the strain to the antibiotic (Figure 10D).

#### 3.9.3. Synergistic Evaluation of *T. subulata* Extract Combined with an Antibiotic

**Figure 11:**
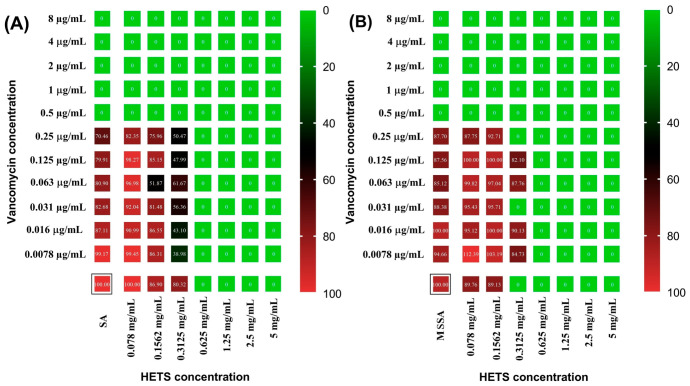
Heat map of the antibacterial activity of the HETS extract associated with vancomycin against *S. aureus* ATCC 6538 (**A**) and *S. aureus* ATCC 25923-MSSA (**B**). The color gradient indicates the metabolic activity of the bacteria, where red indicates high metabolic activity and green indicates low metabolic activity. The horizontal axis shows the concentrations of the HETS extract (0.078–50 mg/mL). The vertical axis shows the concentrations of vancomycin (0.0078–8.0 µg/mL). The results were presented as averages, with n = 2.

The combination matrix between HETS extract and vancomycin for *S. aureus* ATCC 6538 showed no alteration in the inhibition profile when the agents were combined (Figure 11A). For concentrations below the MIC of HETS extract (0.078–0.625 mg/mL), metabolic activity remained elevated, ranging from 38.8% to >90%, independent of the vancomycin concentration. From 0.6 mg/mL, the extract maintained complete inhibition of metabolic activity, and this pattern remained unchanged in all combinations with the antibiotic.

In Figure 11B, a similar matrix was observed for the *S. aureus* ATCC 25923-MSSA strain. At subinhibitory concentrations of the HETS extract (0.078–0.6 mg/mL), metabolic activity values remained elevated, above 82.10%, except for isolated situations at the 0.3 mg/mL extract concentration, where complete inhibition of metabolic activity was observed. This was not attributed to any antibacterial activity, but to possible interferences in the assay. As with the *S. aureus* ATCC 6538 strain, the HETS extract resulted in complete growth inhibition from 0.6 mg/mL, and this response remained unchanged in the matrix, without modulation by the antibiotic.

When comparing the two strains (Figure 11), it became evident that both presented the same response pattern, with no further reduction in the metabolic activity of the bacteria at the combination points and maintenance of values equivalent to those observed for each isolated agent. For both strains, no regions of the matrix indicating altered bacterial susceptibility were identified, and the transition from high metabolic activity to complete inhibition occurred exclusively as a function of the increased concentration of the extract and the antibiotic. Consistently, FICI (Fractional Inhibitory Concentration Index) values of 2.0 were obtained for both strains, indicating an indifferent interaction between HETS and vancomycin.

It is important to note that MIC values obtained for vancomycin in this study were based on XTT metabolic activity measurements rather than standard turbidity endpoints. This methodological difference may result in higher apparent MIC values, as residual metabolic activity can persist despite effective growth inhibition.

## 4. Discussion

This study evaluated the cytotoxic, antioxidant, immunomodulatory, and antimicrobial properties of aqueous (AETS) and hydroethanolic (HETS) extracts of *T. subulata*. The results presented in this study complement previous reports of the biological activity of *T. subulata* extracts [3,4], emphasizing the influence of the extraction solvent on the physicochemical characteristics, biochemical composition, and biological performance of the aerial parts of this species.

Although previous studies have reported the biological potential of *T. subulata*, including antioxidant, anti-inflammatory, and antimicrobial activities [3,4,5,7,8], direct quantitative comparison with the present results remains limited due to methodological differences, such as the use of specific plant parts instead of aerial parts, variations in extraction procedures, and distinct experimental conditions across assays. For instance, antioxidant activity reported for *Turnera* species shows high radical scavenging capacity [42,43], but under different concentration ranges and protocols, which restricts direct comparison of absolute values. Similarly, antimicrobial activity against *S. aureus* has been described [7,8], although using different extract preparations. Despite these limitations, our results are consistent with general trends in the literature, particularly regarding the influence of solvent polarity on phenolic extraction [5]. Therefore, the integrated and standardized approach adopted in this study, combining multiple biological assays under controlled conditions, advances the characterization of *T. subulata* and highlights the need for harmonized methodologies in future studies to enable more robust comparisons.

The LC–MS/MS analysis revealed a consistent chemical composition between extracts, with eleven compounds detected in both, including organic acids (gluconic and citric acids), phenolic acids (gentisic acid hexoside, feruloylquinic acid, and O-caffeoylquinic acid), and predominantly flavonoids, especially C- and O-glycosylated apigenin derivatives such as homoorientin, vitexin, vitexin-O-hexosides, apiin, and apigenin-O-hexoside. These results indicate that the differences in biological activity are not related to the presence or absence of compounds, but rather to their relative abundance, which is modulated by solvent polarity. These results are aligned with the study by Luz et al. [5], demonstrating that solvent polarity modulates the yield and bioactivity of secondary metabolites of plant origin. Additionally, variations in phytochemical composition may be influenced by environmental and geographical factors, such as climate, soil characteristics, and biotic stress, which can affect the biosynthesis of secondary metabolites in *T. subulata*.

The significantly higher content of phenolic compounds observed in HETS compared to the AETS extract is consistent with the intermediate polarity of 40% ethanol, which favors the co-extraction of both polar and moderately non-polar phenols, including flavonoids and phenolic acids previously identified in *T. subulata*, such as vitexin-2-O-rhamnoside and ferulic acid derivatives [4,5]. The predominance of these metabolite classes, as verified by chromatographic screening, provides a plausible chemical basis for the enhanced antioxidant and antimicrobial responses observed for HETS, translating into a greater capacity to scavenge radicals in the DPPH and ABTS assays, in which HETS achieved inhibition values (>83%) comparable to those reported for other species, such as *T. ulmifolia* and *T. aphrodisiaca* at similar concentrations [42,43]. However, when evaluated by the TRAP assay, which quantifies the ability of antioxidants to retard fluorescence decay in a probe under controlled peroxidative conditions initiated by ROO• generated by AAPH, a model closer to physiological lipid peroxidation, the antioxidant capacity showed the opposite behavior [44]. AETS showed a significant reduction in AUC starting at 50 µg/mL, whereas HETS did not demonstrate significant inhibition at concentrations above 50 µg/mL and displayed a pro-oxidant effect at lower doses. This behavior likely reflects the redox complexity of phenol-rich matrices, in which auto-oxidation of polyphenols or interactions with trace metal ions can promote pro-oxidant effects, as previously documented for several plant extracts [45,46].

The extracts did not inhibit the degradation of 2-deoxyribose mediated by hydroxyl radicals; instead, a dose-dependent pro-oxidant behavior was observed in this system based on the Fenton reaction. This pro-oxidant pattern suggests an intrinsic redox-active property of the extracts or specific artifacts inherent to the 2-DR assay. This may occur due to the sensitivity of the deoxyribose method to the iron redox cycle, secondary formation of Fe^3+^, and potential auto-oxidation of phenolic constituents [47,48]. Previous studies highlight the need for kinetic assays, optimized blanks for Fe^3+^, and complementary tests to reliably distinguish true pro-oxidant effects from analytical interference [47,49,50]. The presence of multiple phenolic subclasses, inferred from exploratory chemical profiling, likely contributes to these context-dependent redox effects, reinforcing the limitations of single in vitro antioxidant models.

Furthermore, in plant extracts, phenolic compounds and flavonoids are known to modulate Fenton chemistry and potentially amplify the generation of reactive oxygen species under high availability of metal ions. Studies such as those conducted by Nowak et al. [46] and Shin et al. [48] reported a dose-dependent trade-off between antioxidant and pro-oxidant activity due to OH generation or auto-oxidation of extract components. These studies support the hypothesis that both the phenolic profile and metal ion interactions play key roles in in vitro oxidative models. Furthermore, the context-dependent results of this study reinforce the limitations of isolated in vitro antioxidant assays and the need for multiple methodological approaches.

Cytotoxicity assessments in SH-SY5Y neuroblastoma cells showed a clearly solvent-dependent profile, with HETS exhibiting the greatest reduction in viability from 50 µg/mL. This result is attributed to the higher phenolic load of HETS, which, at high concentrations, can exhibit cytotoxic effects [51]. These different compounds between the HETS and AETS extracts can induce cell death through mechanisms such as modulation of oxidative stress or induction of apoptosis, which are more pronounced at moderate concentrations [52]. In contrast, AETS maintained safer cell viability up to 500 µg/mL, indicating that its more polar components, such as glycosylated polysaccharides and flavonoids, previously reported in the literature, are less likely to cause cell death [5].

An atypical response was observed at the highest concentration of the HETS extract tested (5000 µg/mL), where the percentage of MTT reduction did not differ from the control group. This behavior may reflect the intrinsic limitations of the colorimetric assay, since MTT reduction does not exclusively assess the number of viable cells, but also the intensity of metabolic activity, mainly dependent on NAD(P)H and mitochondrial dehydrogenases [53]. Thus, metabolic hyperstimulation of remaining cells, frequently induced by intense stress, can result in increased production of reducing equivalents, raising MTT conversion without reflecting a greater number of viable cells [54]. Furthermore, direct chemical interactions between compounds present in the extract and the tetrazolium reagent, as well as the possible release of reducing metabolites after cell death, may contribute to this artificial increase in the signal [55]. These results reinforce the need for validation by complementary methods.

The HETS extract was selected for immunomodulation assays in splenocytes and for antimicrobial evaluation based on objective criteria. HETS showed (i) a significantly higher total phenolic compound content than AETS (*p* < 0.0001), (ii) greater antioxidant activity in the DPPH and ABTS stable radical scavenging assays (up to 85% inhibition), and (iii) despite greater cytotoxicity in SH-SY5Y cells, it is understood that the HETS extract has a higher and more diverse concentration of bioactive metabolites of interest. Since the literature demonstrates that the immunomodulatory and antimicrobial activities of species of the genus Turnera are mainly attributed to flavonoids and phenolic derivatives [1,4,5,29], the extract with the highest concentration of phenolic compounds and constituents was chosen to maximize the probability of detecting relevant biological effects, optimizing experimental resources, and avoiding dilution of response with the extract of lower phytochemical content.

Thus, based on the results of the MTT and SRB assays, a limit of 50 µg/mL was established for conducting the assays in splenocytes. This dose represents the experimentally observed inflection point, where the HETS extract maintained a higher observation at comparable doses. Therefore, a maximum exposure of 50 µg/mL to primary immune cells (T cells, B cells, macrophages, and dendritic cells that make up the splenocyte population) ensures that splenocyte experiments examine the upper limit of the subtoxic range found in previous assays, while maintaining a conservative margin to avoid confusion between immunomodulation and cytotoxicity [56,57].

The immunomodulatory potential of HETS was evidenced by the significant increase in IL-10 in splenocytes at non-cytotoxic concentrations (12.5 µg/mL). This effect is consistent with the known immunomodulatory properties of flavonoids such as vitexin, apigenin derivatives, and homoorientin, which can modulate signaling pathways including NF-κB and MAPK, promoting an anti-inflammatory profile [58,59]. The selective increase in IL-10 suggests a balance in Th1/Th2/Th17 responses, a pattern associated with the resolution phase of inflammation and potentially beneficial in chronic neurodegenerative or autoimmune conditions [60].

Antimicrobial evaluation revealed selective bacterial growth inhibition by HETS against *S. aureus*, including an MSSA strain, with MIC values of 0.625–1.25 mg/mL, while Gram-negative strains of *E. coli* were virtually unchanged. This selectivity for Gram-positive bacteria is a common characteristic of phenol-rich extracts and is attributed to the absence of an outer membrane and the greater affinity of phenols for the peptidoglycan-rich cell wall [61,62]. Although the MIC values of crude extracts are typically higher than those of pure antibiotics, the activity observed here compares favorably with other medicinal plants against MSSA [63]. The absence of synergistic interaction with vancomycin indicates independent mechanisms of action, likely involving membrane disruption and enzyme inhibition by phenols, rather than interference with the synthesis of the target cell wall of glycopeptides [64,65,66].

In general, the solvent-dependent bioactivity profile of *T. subulata* corroborates the traditional use of hydroethanolic preparations in Brazilian folk medicine and provides mechanistic insights into their antioxidant, immunomodulatory, and antistaphylococcal properties. Although selective cytotoxic effects in neuronal cells were observed at higher concentrations, together with non-cytotoxic immunomodulatory responses, these findings should be interpreted cautiously and do not allow direct extrapolation to therapeutic applications or disease contexts, including neurodegenerative disorders.

Taken together, the present findings indicate that the extraction solvent substantially shapes the in vitro biological profile of *T. subulata*, with the hydroethanolic extract concentrating phenolic constituents and showing the most consistent activity across redox, cytokine, and antibacterial assays. However, these results should be interpreted within the limits of the study, which relied on crude extracts, exploratory chemical profiling, cytokine assessment at a single non-cytotoxic concentration, and in vitro models only. Therefore, the current data support solvent-dependent redox behavior, cytokine-modulating effects in murine splenocytes, and selective anti-staphylococcal activity under the tested conditions, rather than broader anti-inflammatory, neuroprotective, or therapeutic claims. Future studies should combine full chemical annotation, bioassay-guided fractionation, and mechanistic validation to identify the constituents responsible for the observed effects and clarify their biological relevance.

Despite the biological activities observed, several technical challenges remain before the potential translation of *T. subulata* extracts into commercial or medical applications. These include the need for standardization of extract composition, identification and isolation of active compounds, assessment of stability and bioavailability, and validation of efficacy and safety in in vivo models. In addition, further studies are required to elucidate the mechanisms underlying the antibacterial activity, including membrane disruption, oxidative stress induction, and interaction with microbial targets. The scale-up of extraction processes and evaluation of regulatory requirements are also essential steps for industrial application. Future research should address these aspects to support the development of safe, effective, and reproducible phytopharmaceutical or nutraceutical products.

## 5. Conclusions

The extraction solvent strongly influenced the in vitro bioactivity of *T. subulata* aerial part extracts, while both extracts presented a similar chemical profile composed mainly of organic acids, phenolic acids, and flavonoids, especially glycosylated derivatives of apigenin putatively annotated by LC–MS/MS. The main difference between the extracts was related to the relative abundance of these compounds rather than their presence or absence. The hydroethanolic extract exhibited higher phenolic compounds and stronger DPPH and ABTS radical scavenging than the aqueous extract, whereas the aqueous extract performed better in the TRAP assay. Both extracts also displayed pro-oxidant effects in the deoxyribose/Fenton system. In cellular assays, the hydroethanolic extract showed the most pronounced biological effects, including dose-dependent cytotoxicity in SH-SY5Y cells, induction of IL-10 production in murine splenocytes at non-cytotoxic concentrations, and selective antibacterial activity against *S. aureus*, with no observed synergism with vancomycin. Overall, these findings suggest that variations in biological responses may be associated with differences in the relative abundance of shared compounds influenced by extraction conditions, highlighting the hydroethanolic extract as a promising source of bioactive molecules for future investigations, although its effects remain context-dependent.

## Figures and Tables

**Table 1:** Physicochemical and biochemical characteristics and extraction yield of aqueous (AETS) and hydroethanolic (HETS) extracts from the aerial parts of *T. subulata*. Data are expressed as mean ± standard deviation. Extraction yield (%) was calculated based on the total solids content of the plant material and the respective extracts.

Parameters	Extracts	
	AETS	HETS
Total solids content (%)	1.14 ± 0.05 *	2.40 ± 0.02 *
Reducing carbohydrates (mg glucose/100 mL)	15.83 ± 0.14 *	2.33 ± 0.01 *
Protein (mg BSA/100 mL)	3.36 ± 0.02 *	16.88 ± 0.06 *
Phenolic compounds (mg GAE/100 mL)	1269.54 ± 20.60 *	2555.96 ± 43.55 *
Extraction yield (%)	25.93 ± 1.40% *	54.50 ± 1.79% *

Note: AETS—aqueous extract of *T. subulata*, HETS—hydroethanolic extract of *T. subulata*. Statistical analysis was performed using one-way ANOVA with Sidak’s multiple comparisons. Significance values were represented as (*) for a significance of *p* < 0.0001.

**Table 2:** Compounds annotated by LC–MS/MS for *T. subulata* extracts, based on data analysis in MS-DIAL software (version 5.5), using reference spectra from the GNPS2 and MS/MS Public Library libraries.

Peak	tR (min)	MS (m/z)	Formula	Putative ID	Reference
1	4.173	195 [M-H]^-^	C_6_H_12_O_7_	Gluconic acid	[29]
2	5.881	191 [M-H]^-^	C_6_H_8_O_7_	Citric acid	[30]
3	8.583	315 [M-H]^-^	C_13_H_16_O_9_	Gentisic acid-O-hexoside	[31]
4	10.930	367 [M-H]^-^	C_17_H_20_O_9_	Feruloylquinic acid	[32]
5	10.989	353 [M-H]^-^	C_16_H_18_O_9_	O-caffeoylquinic acid	[33]
6	13.127	447 [M-H]^-^	C_21_H_20_O_11_	Homoorientin	[34]
7	13.642	593 [M-H]^-^	C_27_H_30_O_15_	Vitexin-O-hexoside I	[35]
8	13.711	577 [M-H]^-^	C_27_H_30_O_14_	Vitexin-O-hexoside II	[36]
9	13.801	563 [M-H]^-^	C_26_H_28_O_14_	Apiin	[37]
10	14.157	431 [M-H]^-^	C_21_H_20_O_10_	Vitexin	[38]
11	15.286	431 [M-H]^-^	C_21_H_20_O_10_	Apigenin-O-hexoside	[39]

## Data Availability

The original contributions presented in this study are included in the article. Further inquiries can be directed to the corresponding authors.

## Abbreviations

2-DR	2-deoxyribose
AAPH	2,2′-azobis-2-methyl-propanamide
ABTS	2,2-azino-bis(3-ethylbenzo-thiazoline-6-sulfonic acid
AETS	Aqueous extract of *T. subulata*
ATCC	American Type Culture Collection
BHI	Brain Heart Infusion
BSA	Bovine serum albumin
CAPES	Coordination for the Improvement of Higher Education Personnel
CLSI	Clinical and Laboratory Standards Institute
CNPq	National Council for Scientific and Technological Development
DMEM	Dulbecco’s Modified Eagle Medium
DNS	3,5-dinitrosalicylic acid
DPPH	2,2-diphenyl-1-picrylhydrazyl
FAPESQ	Research Support Foundation of the State of Paraíba
HETS	Hydroethanolic extract of *T. subulata*
IL-10	Interleukin 10
IL-6	Interleukin 6
MDA	Malondialdehyde
MIC	Minimum inhibitory concentration
MSSA	Methicillin-sensitive *Staphylococcus aureus*
MTT	3-[4,5-dimethylthiazol-2-yl]-2,5 diphenyl-tetrazolium
MV	Plant material
RPMI	Roswell Park Memorial Institute
SRB	Sulforodamine B
TBA	Thiobarbituric acid
TNF-α	Tumor necrosis factor alpha
TRAP	Total antioxidant activity
TRIS	Tris(hydroxymethyl)aminomethane
UEPB	State University of Paraíba
UFCG	Federal University of Campina Grande
UFPE	Federal University of Pernambuco
UFRGS	Federal University of Rio Grande do Sul
XTT	2,3-bis-(2-methoxy-4-nitro-5-sulfophenyl)-2H-tetrazolium-5-carboxanilide)
